# Risk factors for new-onset delirium in patients with bloodstream infections: independent and quantitative effect of catheters and drainages—a four-year cohort study

**DOI:** 10.1186/s13613-016-0205-x

**Published:** 2016-10-28

**Authors:** Tolga Dittrich, Sarah Tschudin-Sutter, Andreas F. Widmer, Stephan Rüegg, Stephan Marsch, Raoul Sutter

**Affiliations:** 1 University of Basel, Basel, Switzerland; 2 Division of Infectious Diseases and Hospital Epidemiology, University Hospital Basel, Basel, Switzerland; 3 Division of Clinical Neurophysiology, Department of Neurology, University Hospital Basel, Basel, Switzerland; 4 Clinic for Intensive Care Medicine, University Hospital Basel, Basel, Switzerland

**Keywords:** Bloodstream infections, Catheters, Delirium, Drainages, Risk factors

## Abstract

**Background:**

Bloodstream infections (BSI) and delirium are frequent in critically ill patients. During systemic inflammatory response to BSI, cytokines may interact with neurotransmitters and neuronal receptors driving acute brain dysfunction. However, prospectively collected data on incidence, prediction and impact of delirium in association with BSI are lacking. This study aimed to determine the incidence and predictors of new-onset delirium and its impact on outcome in critically ill adult patients with BSI.

**Methods:**

From 2011 to 2014, all consecutive adult patients with BSI treated in the intensive care units of an academic medical care center were identified. Pertinent clinical and microbiological data including the Intensive Care Delirium Screening Checklist (ICDSC) were assessed. Multivariable analysis was performed to identify variables independently associated with ICDSC ≥4.

**Results:**

Among 240 patients, 145 (60%) had an ICDSC ≥4 (i.e., delirium). In-hospital mortality was 34%. Delirious patients had a higher mortality (40 vs. 23%; *p* = 0.005), a lower proportion with return to functional baseline (30 vs. 46%; *p* = 0.012), and a higher proportion with unfavorable outcome in survivors (74 vs. 54%; *p* = 0.010). Multivariable analyses revealed age (OR 1.04, 95% CI 1.02–1.06), male gender (OR 2.26, 95% CI 1.17–4.36), and the number of catheters and drainages before diagnosis of BSI (OR for every additional catheter = 1.14, 95% CI 1.04–1.25) as independent predictors for delirium (adjusted for SAPS [simplified acute physiology score] II, Riker Sedation-Agitation Scale [SAS], Sequential Organ Failure Assessment [SOFA] score, dementia and/or leukoencephalopathy, and albumin levels).

**Conclusions:**

The incidence of delirium in patients with BSI is high and associated with adverse outcome. The number of catheters and drainages may constitute a useful and readily available predictor of delirium in patients with BSI allowing to identify patients at high risk. Ultimately, reliable identification of patients at increased risk for delirium is key for allocation of specific prevention strategies.

## Background

Sepsis and delirium are frequent and associated with high morbidity and mortality in critically ill patients treated in intensive care units (ICUs) [1, 2]. Sepsis affects more than 25% [3–5], and delirium is present in as many as 80% of mechanically ventilated patients [6–8] and in up to 50% of non-mechanically ventilated patients [9–11]. They often concur, and a number of recent studies provide evidence of complex underlying mechanisms explaining how systemic inflammatory response drives acute brain dysfunction, possibly explaining this association. Systemic inflammation may disturb the integrity of the central nervous system by an increased production and release of cytokines interacting with the somatic autonomic nerve fibers. Associated changes in brain perfusion, and increased diffusion of cytokines through the loosened blood–brain barrier mainly in the circumventricular areas, the choroid plexus [12], or through saturable transport mechanisms [13] may further fuel these interactions. In addition, anorexia, lethargy, and depression in temporal association with systemic inflammation and fever, collectively named sickness behavior, are observed as a response of neurons to cytokines in different animal models [14, 15] and humans [16]. Although it seems evident that activation of the immune system can induce acute brain dysfunction and that sepsis, therefore, is a major risk factor for delirium, most studies are hampered by the fact that sepsis encompasses a variety of different sources and states of infection and do not focus on the immediate and acute neurological impact of specific infections in critical care settings. Although bloodstream infections (BSI) belong to the most important, frequent, and clearly defined infections encountered in ICUs, prospective studies regarding the incidence, prediction, and impact of delirium in association with BSI are not yet published.

We therefore sought to determine incidence and predictors of new-onset delirium and its impact on outcome in critically ill adult patients with BSI.

## Methods

This study was performed in the ICUs of the University Hospital Basel, Switzerland, an academic tertiary medical care center treating more than 47,000 emergencies and with more than 35,000 admissions per year. Patients were derived from an ongoing prospective cohort of patients with BSI [17].

The study was approved by the local ethics committee (Ethikkommission Nordwest-und Zentralschweiz, Nr. 2014/165) according to the declaration of Helsinki, and patients’ consent was waived.

### Demographics and clinical characteristics

From 2011 to 2014, all consecutive adult patients with BSI treated in the medical or surgical ICUs of an academic medical care center were identified. Patients without consecutive ICDSC scoring or with delirium prior to the diagnosis of BSI were excluded. We assessed pertinent clinical, laboratory, and microbiological data including principal diagnoses, the SAPS II scores, comorbidities, transient episodes of coma on the day of BSI diagnosis, and serum levels of acute-phase proteins as described below. Treatment characteristics of all patients were assessed including ICU and hospital stay, duration of mechanical ventilation, the number of catheters and drainages, the use of anesthetics and sedatives during the three days prior to BSI, and the administration of anesthetics and neuroleptic drugs during the three days following BSI. The Intensive Care Delirium Screening Checklist (ICDSC) and outcomes were assessed as mentioned below.

### Sedation protocol

In all patients treated with sedatives, sedation was managed and titrated by using the Riker Sedation-Agitation Scale (SAS). In all patients treated with sedatives, daily interruption of sedation was an integral component of the sedation protocol. To establish sedation, short active sedatives such as disoprivan were preferred providing absence of hemodynamic instability. In patients with hemodynamic instability, sedation was achieved by administration of benzodiazepines given at the lowest possible dose and titrated according to the sedation level as determined by SAS.

### Definition and detection of infections and BSIs

A protocol for monitoring infections was established for all patients in the ICUs during the entire study period. It included drawing cultures of blood and urine, cultures from tracheal aspirates or sputum, and performance of a chest X-ray in any patient with new onset of fever or hypothermia. If other foci were suspected, these were sampled accordingly. Infections were diagnosed based on the patients’ clinical examination, radiological exams, laboratory findings, and microbiological results according to the Centers for Disease Control and Prevention (CDC) criteria [18]. The diagnosis of BSI was defined according to the current guidelines [19] and confirmed by two infectious diseases specialists in patients with at least one positive blood culture and concurrent presence of the systemic inflammatory response syndrome. Systemic inflammatory response was diagnosed if two or more of the following criteria were fulfilled: body temperature >38 or <36 °C; heart rate >90/min; respiratory rate >20/min; serum leukocyte count >12 or <4 G/L or the presence of >10% immature neutrophil granulocytes in the blood samples. Catheter-related BSI was defined according to the CDC criteria (www.cdc.gov/hai/bsi/bsi.html).

New definitions for sepsis and septic shock were published in 2016 [20], recommending to define sepsis as life-threatening organ dysfunction, captured by the Sequential [Sepsis-related] Organ Failure Assessment (SOFA) score, and defining septic shock as the need for vasopressors to maintain a mean arterial blood pressure of ≥65 mmHg and serum lactate level >2 mmol/L in the absence of hypovolemia. Therefore, we retrospectively collected the respective information to calculate the SOFA score and to identify septic shock accordingly.

### Definition and detection of new-onset delirium

Among several screening methods to detect delirium in ICUs, the Confusion Assessment Method for the ICU (CAM-ICU) [6] and the Intensive Care Delirium Screening Checklist (ICDSC) [9] have been most frequently employed. Both have been recommended for the screening of delirium in ICUs by the Society of Critical Care Medicine based on high-quality evidence. [21] Direct comparisons of the diagnostic accuracy of the CAM-ICU and the ICDSC have been performed in recent studies with heterogenous ICU populations revealing a higher sensitivity and specificity of the ICDSC than the CAM-ICU [22–24].

Based on these data, in our institution, the ICDSC is daily assessed in all ICU patients. For the present study, the ICDSC scores on the day before, the day of, and the day following diagnosis of BSI were used. To reduce the interference of sedation in mechanically ventilated patients, ICDSC assessments were performed in mechanically ventilated patients after routine daily interruption of sedation at our institution.

According to the studies and guidelines mentioned above, an ICDSC ≥4 was defined as delirium [9, 21].

### Measurements of acute-phase proteins

For the present study, serum levels of acute-phase proteins (including CRP [C-reactive protein] and albumin), measured daily in all consecutive patients during the study period, were included on the day before, the day of, and the day following the diagnosis of BSI. CRP concentrations were determined by an enzyme immunoassay with a detection limit of 0.5 mg/l (EMIT; Merck Diagnostica, Switzerland). Values <10 mg/l are considered as normal.

### Outcomes

The primary outcome was the development of delirium ±24 h around the diagnosis of BSI. Secondary outcomes were death during the same hospital stay, and return to functional baseline and unfavorable outcome in survivors defined as a Glasgow Outcome Score (GOS) of 1–3 at discharge.

### Statistics

The Chi-square and Fisher exact test (where appropriate) were used for comparisons of proportions. For continuous variables, the Shapiro–Wilk test was used to distinguish between normal and abnormal distributions. Normally distributed variables were analyzed using the Student’s *t* test and non-normally distributed variables using the Mann–Whitney *U* test. To address possible confounding, all variables found to be significant in univariable analyses and known risk factors (i.e., SAPS II, dementia/leukoencephalopathy) were included in the multivariable regression models after calculating odds ratios by logistic regression. In addition, SAS and SOFA were included into the multivariable logistic regression model to correct for the effects of sedation and organ failure on ICDSC scores [25]. Stepwise logistic regression using stepwise forward and backward selection, as well as Akaike information criterion (AIC), was applied to identify variables independently associated with ICDSC ≥4, and collinearity between covariates was assessed.

The Hosmer–Lemeshow goodness-of-fit test was applied to check the final models. Areas under the receiver operating characteristic (ROC) curve were calculated for the final models to evaluate discrimination.

Two-sided *p* values ≤0.05 were considered statistically significant. Statistical analyses were performed with STATA^®^ version 13.0 (Stata Corp., College Station, TX).

## Results

A total of 309 patients with BSI fulfilled the study criteria: 69 patients without consecutive ICDSC scores on all three defined days or with delirium prior to the diagnosis of BSI were excluded. There was no significant difference regarding all baseline characteristics, complications, and outcomes between the excluded and included patients. In the remaining 240 patients, median age was 68 years (interquartile range [IQR] 58–77), median ICU stay was 7 days (IQR 3–19), and median hospital stay 28 days (IQR 15–54). During intensive care, 141 patients (58.8%) were mechanically ventilated for a median of 8 days (IQR 3–19) and the median number of catheters and drainages per patient was 6 (IQR 4–9).

**Fig. 1:**
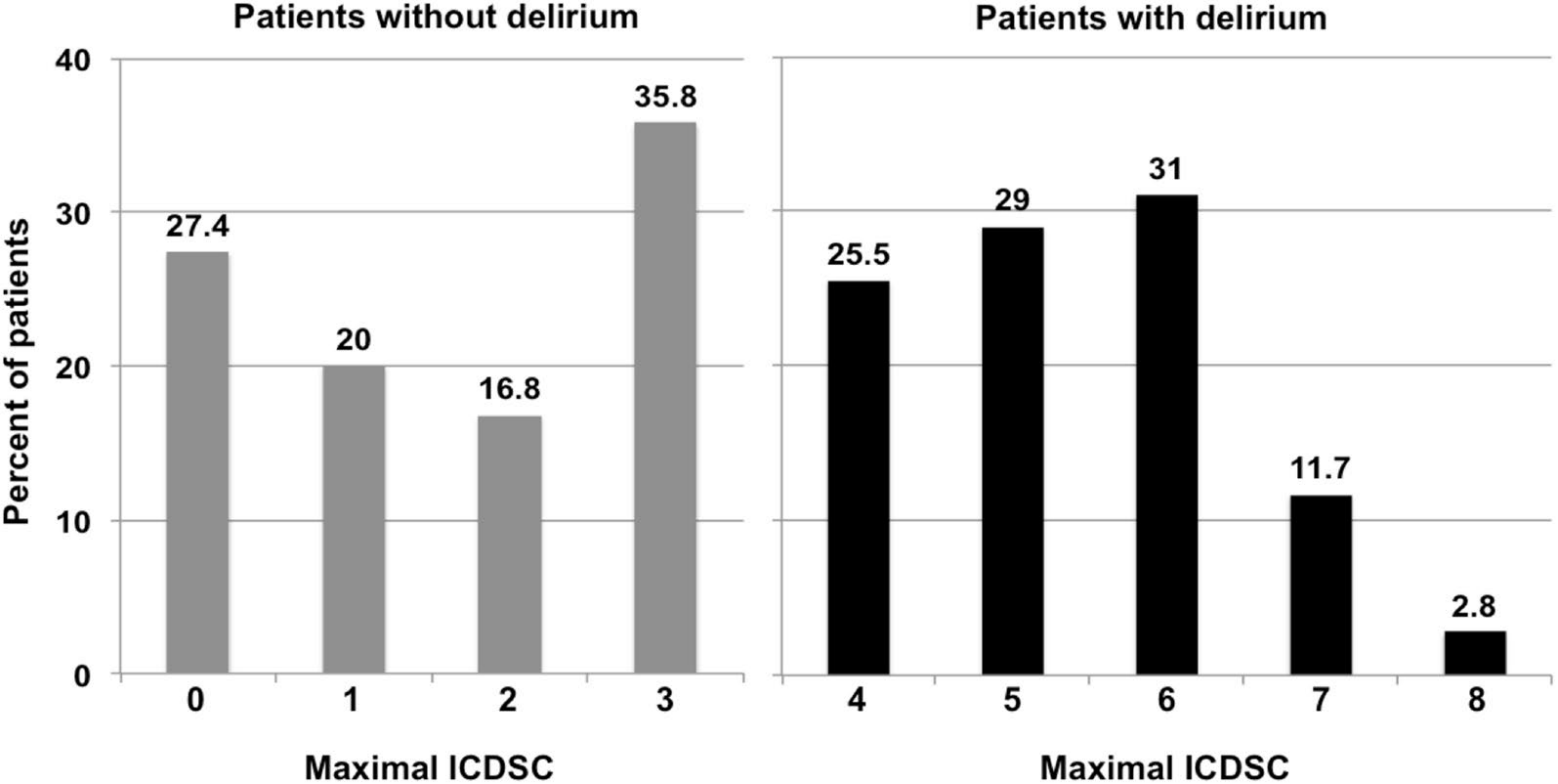
Distribution of the maximal ICDSC ±24 h around diagnosis of bloodstream infections in patients with and without delirium. *ICDSC* the Intensive Care Delirium Screening Checklist

**Fig. 2:**
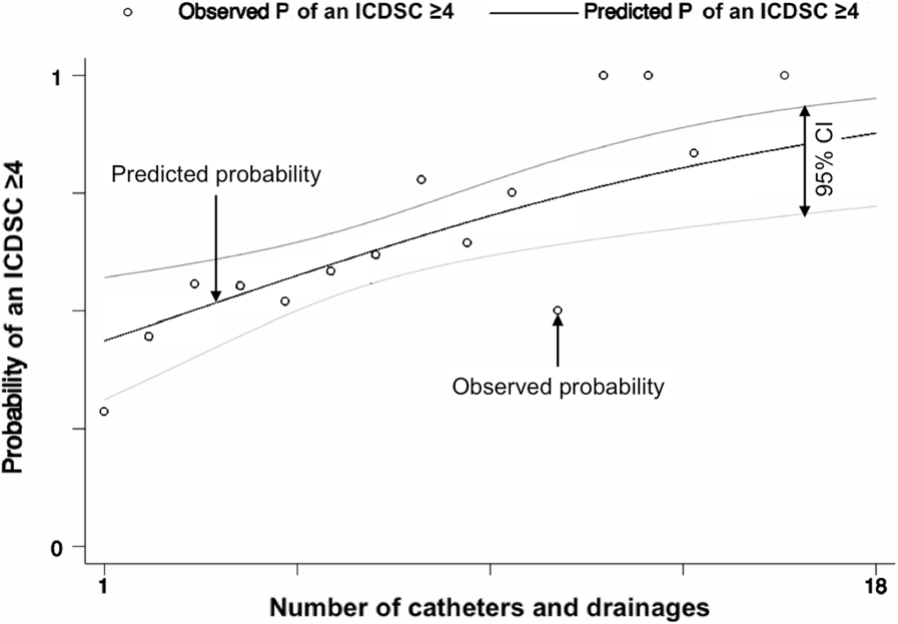
Probability of an ICDSC ≥4 during bloodstream infections in relation to the number of catheters and drainages. *ICDSC* Intensive Care Delirium Screening Checklist, *CI* confidence interval

New-onset delirium, defined as an ICDSC ≥4 ± 24 h around BSI diagnosis, was identified in 145 (60%) patients with BSI. The median duration of new-onset delirium was 4 days (IQR 2–8). The median time from admission to BSI and delirium was 7 days (IQR 2–16) and 8 days (IQR 2–19), respectively. Delirium emerged in most patients immediately before or together with the diagnosis of BSI (in 75 patients [51.7%] on the day before BSI, in 50 patients [34.5%] on the day of BSI diagnosis, and in 20 patients [13.8%] one day following BSI diagnosis). The distribution of the highest ICDSC ±24 h around BSI diagnosis in patients with and without delirium is presented in Fig. 1. In delirious patients, 85.5% had an ICDSC of 4–6. The ICDSC of our patients with a ICDSC <4 was 1–3 in 72.6%, representing a significant proportion of patients with “sub-syndromal delirium.” The use of anesthetics and sedatives three days prior to BSI did not significantly differ between patients with and without an ICDSC ≥4 (40 vs. 35.8%; *p* = 0.512). The univariable comparisons of demographics, baseline characteristics, and details regarding BSIs between patients with BSI with and without new-onset delirium are presented in Tables 1 and 2. Among all patients with BSI, 92 (26.1%) developed septic shock. Serum levels of CRP and leukocytes on the day before, the day of, and the day after BSI diagnosis did not differ between delirious and non-delirious patients. Comparisons of treatment characteristics and outcomes are summarized in Table 3. In delirious patients, mortality was 33.8%. Delirious patients had an almost twofold increased mortality, a lower proportion with return to functional baseline, and a higher proportion with unfavorable outcome in survivors, as compared to non-delirious patients (Table 3). Multivariable analyses revealed that delirium was independently associated with death in our cohort (adjusting for well-established outcome predictors in ICU patients, such as age, SAPSII score, the Charlson Comorbidity Index, and albumin serum levels at admission, OR 1.90, 95% CI 1.01–3.63; *p* = 0.049). In univariable analysis, delirious patients were older, more often male, had lower median albumin serum levels at admission, prolonged ICU stay, and had more catheters and drainages before the diagnosis of BSI (Tables 1, 3). Every additional catheter or drainage increased the probability of delirium (OR_for every catheter/drainage_ = 1.14, 95% CI 1.05–1.24; *p* = 0.002; Fig. 2). The number and duration of mechanical ventilation did not differ between patients with and without delirium (Table 3). The sources of BSI, the distribution of gram-positive and gram-negative pathogens, and the number of infectious complications did not differ significantly between patients with and without delirium (Table 2). Most BSIs were catheter-related not differing between patients with and without delirium.

**Table 1:** Univariable comparisons of baseline characteristics between ICU patients with and without ICDSC ≥4 during bloodstream infections (*n* = 240)

Patient’s characteristics	BSI patients with ICDSC <4 (*n* = 95)		BSI patients with ICDSC ≥4 (*n* = 145)		*p* value
Demographics					
Age (years; median, IQR)	63	48–72	70	60–78	*<0.001*
Female (*n*, %)	40	42.1	39	26.9	*0.014*
Male (*n*, %)	55	57.9	106	73.1	
Clinical features					
SAPS II (median, IQR)	47	34–64	52	38–69	0.070
Main principal diagnoses (*n*, %) not mutual exclusive					
Cardiac disease	58	61.1	103	71.0	0.108
Pulmonary disease (other than ARDS/ALI)	58	61.1	76	52.4	0.188
ARDS/ALI	9	9.5	10	6.9	0.474*
Gastrointestinal tract disease	46	48.4	74	51.0	0.692
Acute ischemic stroke	7	7.4	17	11.7	0.379*
Seizure or status epilepticus	5	5.3	8	5.5	
Intracranial hemorrhage	3	3.2	6	4.1	
Polytrauma	5	5.3	4	2.8	
Traumatic brain injury	4	4.2	2	1.4	
Hypoxic ischemic encephalopathy	2	2.1	3	2.1	
Surgery	50	52.6	81	55.9	0.623
Gastrointestinal tract surgery	19	20.0	17	11.7	0.079
Cardiac surgery	4	4.2	13	9.0	0.203*
Thoracic surgery	2	2.1	7	4.8	
Brain surgery	1	1.1	2	1.4	
Other surgery	24	25.3	42	29.0	0.530
Cardiopulmonary resuscitation	16	16.8	34	23.5	0.218
Comorbidities					
Charlson Comorbidity Index (median, IQR)	2	1–4	3	1–4	0.415
Immunosuppression (*n*, %)	48	50.5	65	44.8	0.387
Liver and/or renal insufficiency (*n*, %)	19	20	42	29	0.119
Drug abuse (*n*, %)	10	10.5	28	19.3	0.068
Remote ischemic stroke (*n*, %)	8	8.4	20	13.8	0.205
Leukoencephalopathy (*n*, %)	3	3.2	10	6.9	0.256
Dementia (*n*, %)	2	2.1	8	5.5	0.323
Epilepsy (*n*, %)	3	3.2	3	2.1	
Remote cerebral hemorrhage (*n*, %)	1	1.1	5	3.5	
Chronic alcohol intake	1	1.1	4	2.8	
Transient episodes of coma on day before BSI diagnosis	17	17.9	38	26.2	0.134
SAS (median, IQR)	3	2–4	3	3–5	0.104
C-reactive protein (mg/l)	*Median*	*IQR*	*Median*	*IQR*	
C-reactive protein on day before BSI diagnosis	113	41–209	121	73–225	0.299
C-reactive protein on day of BSI diagnosis	136	62–239	154	80–267	0.364
C-reactive protein on day after BSI diagnosis	159	82–281	178	103–285	0.409
Leukocytes (×10^9^/l)					
Leukocytes on day before BSI diagnosis	12	8–18	12	8–16	0.862
Leukocytes on day of BSI diagnosis	13	8–18	12	8–18	0.853
Leukocytes on day after BSI diagnosis	12	7–18	13	9–18	0.147
Albumin (g/l)					
Albumin at admission	23	16–28	18	15–23	*0.026*
Albumin on day of BSI diagnosis	21	16–26	18	14–23	*0.045*

*BSI* bloodstream infections, *ICDSC* Intensive Care Delirium Screening Checklist, *MICU* medical intensive care unit; SICU = surgical intensive care unit, ICU intensive care unit, *SAPS* simplified acute physiology score, *ARDS* acute respiratory distress syndrome, *ALI* acute lung injury, *SAS* Riker Sedation-Agitation Scale

* Fisher’s exact test; italic: *p* values are considered significant

**Table 2:** Univariable comparisons of detailed characteristics of bloodstream infections between ICU patients with and without ICDSC ≥4 (*n* = 240)

Patient’s characteristics	BSI patients with ICDSC <4 (*n* = 95)		BSI patients with ICDSC ≥4 (*n* = 145)		*p* value
SOFA score (median, IQR)	6	3–9	7	4–10	0.052
Septic shock (*n*, %)	38	40.0	54	37.2	0.667
Source of BSI (*n*, %)					
Catheters	27	28.4	35	24.1	0.458
Gastrointestinal tract	19	20.0	29	20.0	1.000
Respiratory tract	17	17.9	27	18.6	0.887
Intravascular/endocardial	10	10.5	6	4.1	0.065
Urogenital tract	5	5.3	8	5.5	0.932
Skin/soft tissue	3	3.2	9	6.2	0.373*
Bones	2	2.1	7	4.8	0.489*
Surgical site	0	0.0	3	2.1	
Central nervous system	1	1.0	0	0.0	
Unknown	11	11.6	21	14.5	0.518
Bacteremia pathogens (*n*, %)					
Bacteremia with gram-negative pathogens	44	46.3	58	40.0	0.333
Bacteremia with gram-positive pathogens	51	53.7	87	60.0	
Details/management of catheter-related BSI (*n*, % of catheter-related BSI)					
Exit site infection	4	14.8	3	8.6	0.689*
Catheter removal	25	92.6	33	94.3	1.000*
Infectious complications (*n*, %)					
Ventilator associated pneumonia	1	1.0	6	4.1	0.249*
Catheter-related urinary tract infection	1	1.0	0	0.0	

*BSI* bloodstream infections, *ICDSC* Intensive Care Delirium Screening Checklist, *SOFA score* Sequential [Sepsis-related] Organ Failure Assessment Score

* Fisher’s exact test; italic *p* values are considered significant

**Table 3:** Univariable comparisons of treatment and outcome between ICU patients with and without ICDSC ≥4 during bloodstream infections (*n* = 240)

Treatment and outcome	BSI patients with ICDSC <4 (*n* = 95)		BSI patients with ICDSC ≥4 (*n* = 145)		*p* value
Medical care					
ICU stay in survivors (days; median, IQR)	4	2–11	10	4–24	*0.005*
Hospital stay in survivors (days; median, IQR)	27	16–55	35	21–62	0.224
Mechanical ventilation (*n*, %)	91	62.8	50	52.6	0.119
Mechanical ventilation at BSI onset (*n*, %)	42	44.2	58	40.0	0.518
Duration of mechanical ventilation in survivors (days; median, IQR)	10	3–27	9	3–23	0.799
Number of catheters and drainages (median, IQR)	5	4–7	7	5–10	*0.001*
Treatment with neuroleptic drugs for delirium (*n*, %)	13	13.7	83	57.2	*<0.001*
Treatment with anesthetic drugs for delirium (*n*, %)	48	50.5	100	69.0	*0.004*
Treatment with vasopressors (*n*, %)	38	40.0	54	37.2	0.667
Outcome (*n*, %)					
Death	22	23.2	59	40.7	*0.005*
Return to functional baseline at hospital discharge	44	46.3	44	30.3	*0.012*
GOS 1–3 (unfavorable) in survivors	40	54.8	64	74.4	*0.010*

*BSI* bloodstream infections, *ICDSC* Intensive Care Delirium Screening Checklist, *ICU* intensive care unit, *IQR* interquartile range, *GOS* Glasgow Outcome Score

Italic: *p* values are considered significant

Uni- and multivariable logistic regression analyses including characteristics significantly differing between patients with and without delirium (Tables 1, 3), as well as established risk factors for delirium (i.e., SAPS II, dementia/leukoencephalopathy) and SAS and SOFA scores to correct for the effects of sedation and organ failure on ICDSC are shown in Table 4. All three stepwise selection approaches revealed older age, male gender, and the number of catheters and drainages as predictors for delirium, independent of possible confounders (i.e., age, gender, SAPS II, SAS, SOFA scores, dementia and/or leukoencephalopathy, and albumin serum levels). The mean variance inflation factor for all variables included in the multivariable models was 1.26, ranging from 1.03 to 1.68, ruling out high collinearity and indicating that the selected variables are independent from other predictors. The Hosmer–Lemeshow goodness-of-fit test for the model selection by AIC was *χ* ^2^ = 4.67 (*p* = 0.792) and for the stepwise forward and backward selection was *χ* ^2^ = 9.45 (*p* = 0.306), indicating adequate model fit. The area under the ROC curve for the stepwise regression model was 0.072 supporting its ability to discriminate between patients with and without ICDSC ≥4 during BSI.

**Table 4:** Uni- and multivariable logistic regression analyses of predictors for an ICDSC ≥4 during bloodstream infections (*n* = 240)

Predictors for ICDSC ≥4 during BSI	Univariable			Multivariable* (stepwise model selection by Akaike information criterion)		
	OR	95% CI	*p* value	OR	95% CI	*p* value
Age	1.04	1.02–1.06	*<0.001*	1.04	1.02–1.06	*<0.001*
Male gender	2.00	1.14–3.42	*0.015*	2.26	1.17–4.36	*0.015*
SAPS II	1.01	0.99–1.03	0.063	–	–	–
SAS	1.20	1.01–1.42	*0.041*	1.18	0.96–1.45	0.117
SOFA score	1.06	1.00–1.14	0.059	–	–	–
Dementia and/or leukoencephalopathy	2.23	0.79–6.31	0.130	–	–	–
Albumin at admission (for every increasing mg/L)	0.96	0.92–0.99	*0.016*	–	–	–
Number of catheters and drainages (for every additional catheter)	1.14	1.05–1.24	*0.002*	1.14	1.04–1.25	*0.004*

*BSI* bloodstream infections, *ICDSC* Intensive Care Delirium Screening Checklist, SAPS simplified acute physiology score, *SAS* Riker Sedation-Agitation Scale, *SOFA score* Sequential [Sepsis-related] Organ Failure Assessment Score

* Stepwise forward and backward selection including all variables presented in the table yielded identical results; italic *p* values are considered significant

## Discussion

New-onset delirium in adult ICU patients with BSI is frequent and associated with increased morbidity and mortality. In our study, the incidence of new-onset delirium in close temporal association with BSI diagnosis (60%) is in the upper range of the incidence in the general ICU population (23–65%) [26], suggesting that BSI in critically ill patients may have a promotional effect on the development of delirium. In addition, the ICDSC of our patients with an ICDSC <4 was 1–3 in 72.6%, representing a significant proportion of patients with “sub-syndromal delirium” in close temporal relation to BSI. Similar to published studies, our multivariable analyses confirm that established risk factors for acute brain dysfunction in critically ill patients including older age [26], male gender, and low serum levels of albumin [27] are predictors of new-onset delirium in close temporal association with the diagnosis of BSI. Other well-known predictors for delirium in the general ICU population identified by a recent large systematic review [26] seem to have no significant influence on the development of delirium in the context of BSI, such as altered level of consciousness, or surgery. However, interpretations regarding the association between trauma and delirium in our study are hampered, as patients with trauma are underrepresented in our cohort. The underlying mechanisms of male gender being a predisposing factor for delirium are not fully understood. Our finding that males in our cohort were suffering more often from delirium than females is in line with other studies describing delirium more frequently in males in different clinical settings, such as in surgical ICUs [28] and in elderly patients of general medical and surgical wards [29, 30]. However, other studies of ICU populations did not confirm such an association.

A new finding compared to published studies was the number of catheters and drainages per patient prior to the diagnosis of BSI as another independent predictor of new-onset delirium. At first glance, the odds ratio of 1.14 for delirium by the number of catheters and drainages may seem small. However, it has to be taken into account that they are given for each additional catheter or drainage. Hence, the odds for delirium increases for example with two additional catheters by 28%. Given the inherent limitations of observational data, we attempted to overcome confounding by including all variables known or assumed to impact the emergence of delirium and characteristics differing significantly different between delirious and non-delirious patients in our cohort into the multivariable models. Therefore, the SAPS II score and transient episodes of coma [26] prior to BSI diagnosis were included in our multivariable models. Three different stepwise model selection techniques were applied, all yielding identical results, underlining the robustness of our results. We acknowledge that the association between the number of catheters and drainages and delirium identified in our cohort may still be confounded by unmeasured variables associated with both the use of catheters and drainages and delirium. Our result is, however, supported by other studies identifying use of physical restraints as an important risk factor for the development of delirium [31]. Catheters and drainages preclude mobilization and have been associated with delirium in cardiac surgery patients [32]. Use of restraint was identified as a risk factor for delirium in mechanically ventilated patients [33] and in a multicenter study of ICU patients, which, however, could not assess its independent predictive value [34]. In our cohort, the use of lines was related to delirium despite the fact that all patients were subjected to a systemic inflammatory response triggered by bacteremia—another important contributor to delirium—underscoring its independent contribution.

Strong systemic inflammatory responses can induce acute brain dysfunction. Pro-inflammatory cytokines, particularly interleukin (IL)-1beta and tumor necrosis factor alpha (TNF-alpha), are produced in the periphery, interact with the brain, and initiate cytokine synthesis in the central nervous system [35]. Another major hypotheses trying to explain inflammation-related acute brain dysfunction is the change in neurotransmitters in relation to inflammation. In a study of septic animals, the use of cholinergic agonists improved cognitive performance [36], indicating that cholinergic neurons may be particularly sensitive to systemic inflammation and acute stressors, such as infections, influence the GABA-A complex by altering binding sites and modulating the expression of selective GABA-A receptor subunits [37].

The results of our study and the examples of pathophysiologic aspects outlined above suggest a direct pathomechanistic link between BSI, systemic inflammatory response and acute brain dysfunction expressed as delirium, calling for heightened awareness for new-onset delirium in critically ill patients with BSI. The number of catheters and drainages in ICU patients may constitute a useful and readily available predictor of delirium in patients with BSI allowing to identify patients at high risk. Furthermore, the number of catheters and drainages represents a potential target in the prevention of delirium, as it seems the number of insertion sites is modifiable and likely to further drive delirium in ICU patients. While catheters and drainages are inserted for specific indications and therefore cannot be omitted in most cases, our findings suggest that emphasis should be placed on removing such accesses as soon as possible not only for prevention of catheter-related infections but also for prevention of delirium. In addition, the use of multi-lumen catheters with the aim of reducing the number of insertion sites may be an important consideration in patients at high risk for delirium.

Our cohort is representative of other adult ICU populations, reflected by median age [6, 10, 11, 24, 38], distribution of gender, the SAPS II [24, 38], the duration of delirium [39], the Charlson Comorbidity Index [10, 11, 38], the proportion of patients with mechanical ventilation [10, 24], ICU stay [10, 38] being similar to prior studies of delirium in ICU patients. However, mortality was higher in our cohort as compared to international epidemiological multicenter studies of delirium [10, 38], possibly indicating an intensifying effect on mortality by the concurrence of BSI and the genesis of delirium.

The limitations of this study include the observational single-center design and the restriction of our cohort to the ICUs. Hence, our results are limited to critically ill ICU patients. However, demographics, clinical characteristics, and outcomes in our cohort are similar to those in prior studies of delirium. Our results that traumatic brain injury and dementia were not predictive for the emergence of delirium in BSI patients need to be interpreted with great caution, as there was only a small number of dementia and traumatic brain injury in our cohort. Our analysis can only provide associations and inference regarding causality cannot be drawn. Despite our attempt to overcome confounding by using multivariable models including well-established confounders, unmeasured residual confounding may have occurred. The fact that delirium was diagnosed by using the ICDSC score limits the generalizability or our results to studies and cohorts examined and rated with the same checklist. However, among several screening methods to detect delirium in ICUs, the CAM-ICU scale [6] and the ICDSC [9] have been most frequently employed. Both have been equally recommended for the screening of delirium in ICUs by the Society of Critical Care Medicine Pain, Agitation, and Delirium guidelines based on high-quality evidence [21]. Direct comparisons of the diagnostic accuracy of the CAM-ICU and the ICDSC have been performed in recent studies with heterogenous ICU populations revealing a higher sensitivity and specificity of the ICDSC than the CAM-ICU [22–24]. The indications for the placement of catheters and drainages may be related to the development of delirium in our cohort. However, the exact indication for the use of catheters could not be identified reliably, especially, as in many patients several different indications concur. Due to the retrospective study design, consecutive data regarding cumulative sedative drug administration could not be assessed. However, such information would be critical, as the cumulative drug administration does not represent the actual individual serum concentrations of the drugs over time, as they largely depend on individual factors, such as renal or liver function, and body weight. In addition, individual drug sensitivity would not be addressed. We further acknowledge that despite daily stopping of sedation and the use of short acting anesthetics, a slight remaining sedative effect may have still influenced the ICDSC assessment—a shortcoming that can only be overcome by excluding mechanically ventilated and sedated patients resulting in a highly selected population not representative of general ICU populations.

## Conclusions

The incidence of new-onset delirium in critically ill patients with BSI is high and associated with increased mortality and unfavorable outcome in survivors calling for heightened awareness and rigorous screening for delirium in patients with BSI during intensive care. Older age, male gender, and the number of catheters and drainages are independent predictors of delirium in close temporal association with BSI. The number of catheters and drainages may constitute a useful and readily available predictor of delirium in patients with BSI allowing to identify patients at high risk. Further studies are needed to externally validate our findings. Ultimately, reliable identification of patients at increased risk of delirium is key for allocation of specific prevention strategies.

## Abbreviations

AIC: Akaike information criterion
BSI: bloodstream infections
CAM-ICU: Confusion Assessment Method for the ICU
CDC: Centers for Disease Control and Prevention
CI: confidence interval
CRP: C-reactive protein
GOS: Glasgow Outcome Score
ICDSC: Intensive Care Delirium Screening Checklist
ICU: intensive care unit
IQR: interquartile range
OR: odds ratio
ROC: receiver operating characteristic
SAPS: simplified acute physiology score
